# Induction of Antimicrobial Resistance in *Escherichia coli* and Non-Typhoidal *Salmonella* Strains after Adaptation to Disinfectant Commonly Used on Farms in Vietnam

**DOI:** 10.3390/antibiotics4040480

**Published:** 2015-10-30

**Authors:** Nguyen T. Nhung, Cao T. Thuy, Nguyen V. Trung, James Campbell, Stephen Baker, Guy Thwaites, Ngo T. Hoa, Juan Carrique-Mas

**Affiliations:** 1 The Hospital for Tropical Diseases, Wellcome Trust Major Overseas Programme, Oxford University Clinical Research Unit, Ho Chi Minh City, Vietnam; E-Mails: thuyct@oucru.org (C.T.T.); trungnv@oucru.org (N.V.T.); jcampbell@oucru.org (J.C.); sbaker@oucru.org (S.B.); gthwaites@oucru.org (G.T.); hoant@oucru.org (N.T.H.); jcarrique-mas@oucru.org (J.C.-M.); 2 Department of Medical Microbiology, Academic Medical Center, University of Amsterdam, Amsterdam 1105 AZ, the Netherlands; 3 Centre for Tropical Medicine, Nuffield Department of Clinical Medicine, Oxford University, Old Road Campus, Roosevelt Drive, Oxford, OX3 7BN, UK

**Keywords:** disinfectant, antimicrobials, adaptation, efflux pumps

## Abstract

In Vietnam, commercial disinfectants containing quaternary ammonium compounds (QACs) are commonly used in pig and poultry farms to maintain hygiene during production. We hypothesized that sustained exposure to sub-bactericidal concentrations of QAC-based disinfectants may result in increased levels of antimicrobial resistance (AMR) among Enterobacteriacea due to the increase of efflux pump expression. To test this hypothesis we exposed six antimicrobial-susceptible *Escherichia coli* (*E. coli*) and six antimicrobial-susceptible non-typhoidal *Salmonella* (NTS) isolates to increasing concentrations of a commonly used commercial disinfectant containing a mix of benzalkonium chloride and glutaraldehyde. Over the 12-day experiment, strains exhibited a significant change in their minimum inhibitory concentration (MIC) of the disinfectant product (mean increase of 31% (SD ± 40)) (*p* = 0.02, paired Wilcoxon test). Increases in MIC for the disinfectant product were strongly correlated with increases in MIC (or decreases in inhibition zone) for all antimicrobials (Pearson’s correlation coefficient 0.71–0.83, all *p* < 0.01). The greatest increases in MIC (or decreases in inhibition zone) were observed for ampicillin, tetracycline, ciprofloxacin, and chloramphenicol, and the smallest for gentamicin, trimethoprim/sulphamethoxazole. The treatment of 155 representative *E. coli* isolates from farmed and wild animals in the Mekong Delta (Vietnam) with phenyl-arginine beta-naphthylamide (PAβN), a generic efflux pump inhibitor, resulted in reductions in the prevalence of AMR ranging from 0.7% to 3.3% in these organisms, indicating a small contribution of efflux pumps on the observed prevalence of AMR on farms. These results suggest that the mass usage of commercial disinfectants, many of which contain QACs, is potentially a contributing factor on the generation and maintenance of AMR in animal production in Vietnam.

## 1. Introduction

Antimicrobial resistance (AMR) is a major global health threat recognized by the research community as well as by international agencies concerned with human and veterinary medicine [1,2]. The intensity of antimicrobial use is firmly established as one of the key drivers for the development of AMR both in human and veterinary medicine [3,4].

Disinfectants are substances applied to kill microorganisms on inanimate objects. In contrast to antimicrobials, which act on specific bacterial targets, disinfectants have multi-factorial mechanisms of action [5]. The use of disinfectants at the end of the food animal production cycle (*i.e.*, terminal cleaning and disinfection) is a crucial procedure for limiting the transmission of infections between animals sequentially housed in the same buildings, therefore contributing to overall flock/herd health and productivity [6]. There has been mounting evidence that exposure to certain disinfectant agents may induce reduced susceptibility and cross-resistance with antimicrobial agents [7,8,9].

Quaternary ammonium compound (QAC)-based disinfectants are some of the most commonly used in industry and in animal production because of their stability and low cytotoxicity, as well as being non-corrosive on farm equipment [10]. The potential cross-resistance between QACs and antimicrobial agents has been a particular concern over recent years [11,12]. The increased expression of multidrug efflux pumps has been proposed to be responsible for decreased susceptibility to both QACs and antimicrobials [13].

Recent studies have shown a high prevalence of AMR among Enterobacteriaceae from Vietnamese livestock and poultry farms [14,15,16]. We hypothesized that disinfectants commonly used on farms may be one factor contributing to AMR due to unintentional dilution after application on occupied buildings and a large proportion of commercial products include QACs in their formulation. To test this hypothesis we exposed 12 pan-susceptible Enterobacteriaceae strains to suboptimal levels of a widely used commercial product containing benzalkonium chloride and glutaraldehyde (Product A) and measured changes in their tolerance to the product as well as changes in susceptibility to a range of important antimicrobial agents. In order to investigate whether generic efflux pump expression was responsible for the observed changes, we treated strains with a generic efflux pump inhibitor and measured the changes in AMR before and after treatment.

## 2. Results

### 2.1. Adaptation of Strains to Product A

We investigated 12 pan-susceptible Enterobacteriacea strains (six *E. coli* and six non-typhoidal *Salmonella* (NTS) strains) before and after *in vitro* exposure to commercial Product A, which contained 150 mg/mL of benzalkonium chloride and 150 mg/mL of glutaraldehyde. After the 12-day exposure period, six of 12 strains increased their MIC for Product A (*i.e.*, μg/mL of both active components). 

An increase was observed for four of six field NTS strains and for two of six field *E. coli* strains. One *E. coli* strain reduced its MIC after the adaptation period (Table 1).

After the adaptation period, the overall mean MIC of Product A (12 strains, three experimental replicates per strain, *i.e.*, 36 observations) increased from 21 μg/mL (SD ± 6) to 27 μg/mL (SD ± 12) (*i.e.*, a 31% (SD ± 40) increase) (*p* = 0.02, paired Wilcoxon test) (Table 1). The observed variability in MIC between experimental replicates was minimal, with a median coefficient of variation of 0% (75% inter-quartile range (IQR) 0–11.3%) and 0% (75% IQR 0–14.7%) for MIC readings before and after the adaptation experiment, respectively.

Strains did not significantly change their MIC after culture in disinfectant-free media (Mueller Hinton (MH) broth at 37 °C), 22 µg/mL (SD ± 5) compared with a baseline average 21 µg/mL (SD ± 6), (*i.e.*, a +5% (SD ± 19) of change) (*p* = 0.19, Wilcoxon test).

### 2.2. Relationship between Changes in MIC for Product A and Changes in Antimicrobial Susceptibility

**Figure 1:**
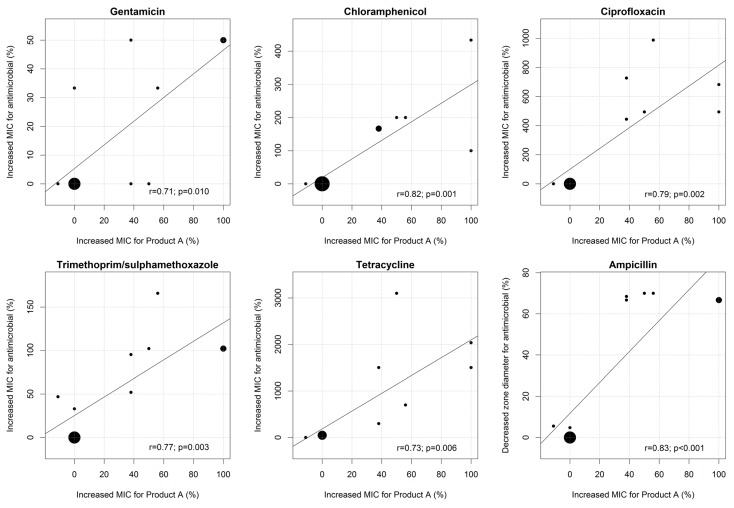
Correlation between changes in MIC for Product A and changes in MIC for gentamicin, chloramphenicol, ciprofloxacin, trimethoprim/sulphamethoxazole, and tetracycline. The size of each dot is proportional to the number of strains.

We observed a strong positive correlation between increased MICs for Product A and increased MICs for gentamicin, chloramphenicol, ciprofloxacin, trimethoprim/sulphamethoxazole, and tetracycline (Pearson’s correlation coefficients (r) ranging from 0.71 to 0.82; all *p* ≤ 0.01). Increases in MIC for Product A were also strongly correlated with reductions in zone diameter in the ampicillin disc diffusion test (*r* = 0.83; *p* < 0.001) (Figure 1).

**Table 1:** Minimum inhibitory concentrations (MIC) against studied strains for Product A. Columns 3, 4, and 6 show MICs (μg/mL) pre-exposure, post-exposure, and after treatment with efflux pump inhibitor (PAβN). Column 5 shows changed MIC levels (%) after exposure to Product A compared with pre-exposure levels. Column 7 shows the average decreased MIC (%) after treatment with efflux pump inhibitor compared with post-exposure levels.

Strain ID	Species	Average MIC (µg/mL)		Average Change in MIC after Exposure	Average MIC (µg/mL)	Average Change in MIC after Treatment with PAβN
		Pre-exposure	Post-exposure		after Treatment with PAβN	
S01	*S.* Derby	21	21	0%	15	−29%
S02	*S.* Ohio	24	33	+38%	24	−27%
S03	*S.* Weltevreden	24	33	+38%	27	−18%
S04	*S.* Weltevreden	30	60	+100%	36	−40%
S05	*S.* Newport	24	36	+50%	30	−17%
SC	*S.* Typhimurium 14028*	24	24	0%	24	0%
E01	*E. coli*	15	15	0%	15	0%
E02	*E. coli*	27	24	−11%	15	−38%
E03	*E. coli*	12	12	0%	12	0%
E04	*E. coli*	12	24	+100%	18	−25%
E05	*E. coli*	27	42	+56%	33	−21%
EC	*E. coli* 25922*	12	12	0%	12	0%
All isolates (mean ± SD)		21 (SD ± 6)	27 (SD ± 12)	+31% (SD ± 40%)	21 (SD ± 9)	−18% (SD ± 15%)

SD: Standard deviation; * Laboratory reference strains.

### 2.3. Changes in Antimicrobial Susceptibility before and after Exposure to Product A

**Figure 2:**
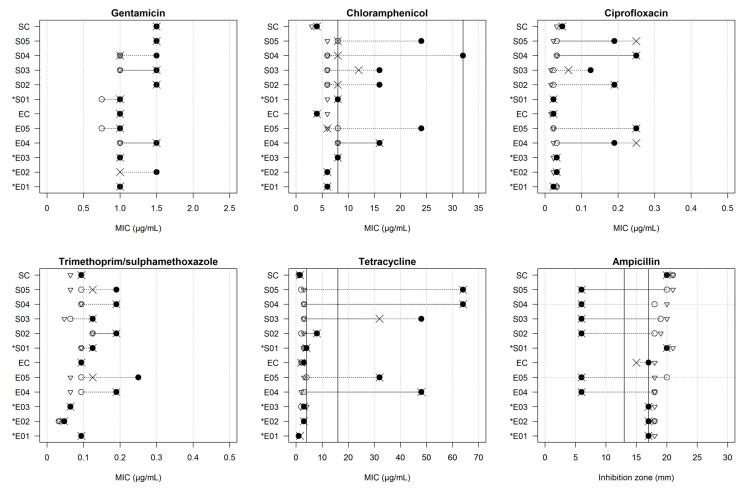
MIC (µg/mL) for antimicrobials of 12 strains before (○) and after (●) exposure experiment; controls of adaptation experiment (∇) and after treatment with efflux pump inhibitor PAβN (x). Vertical black lines: breakpoints for susceptible (on the left), intermediate (between two lines), and resistant (on the right) classes based on Clinical and Laboratory Standard Institute (CLSI) guidelines. For ampicillin, changes in inhibition zone diameter (mm) shown, with full resistance on the left and susceptibility on the right. Breakpoints for gentamicin (≤4 µg/mL, sensitive), ciprofloxacin (≤1 µg/mL, sensitive), and trimethoprim/sulphamethoxazole (≤2 µg/mL, sensitive) not shown since outside the range. * Strains did not increase MIC for Product A.

After exposure to Product A, there were overall MIC increases for all antimicrobials tested (Table 2). The largest changes in MIC were observed for tetracycline (mean change +776%, SD ± 1027), followed by ciprofloxacin (+316%, SD ± 363), chloramphenicol (+106%, SD ± 135), trimethoprim/ sulphamethoxazole (+58%, SD ± 55), and gentamicin (+18%, SD ± 23). The average inhibition zone of ampicillin decreased from 18.6 mm (SD ± 1.5) to 12.0 mm (SD ± 6.4). Qualitative modifications in the AMR profile were demonstrated for tetracycline (three NTS and two *E. coli* isolates developed full resistance and one NTS strain exhibited intermediate resistance), chloramphenicol (four NTS and two *E. coli* isolates exhibited intermediate resistance), and ampicillin (the six strains that showed an increased MIC for Product A also developed ampicillin resistance). After the adaptation experiment, all strains remained susceptible to ciprofloxacin, gentamicin, and trimethoprim/sulphamethoxazole (Table 2 and Figure 2).

**Table 2:** MIC against studied strains for gentamicin, chloramphenicol, ciprofloxacin, trimethoprim/sulphamethoxazole, and tetracycline; zone inhibition diameter for ampicillin before exposure to Product A, after exposure to Product A, and after treatment with efflux pump inhibitor (PAβN). The *p*-values were obtained using paired Wilcoxon tests for the comparisons: ^†^ before and after exposure to Product A; ^§^ after exposure to Product A and after treatment with efflux pump inhibitor.

Antimicrobial	Mean (±SD) (µg/mL)		Mean Change (%) (±SD)	*p*-Value ^†^	Mean (±SD) (µg/mL)	Mean Change (%) (±SD)	*p*-Value ^§^
	Pre-exposure	Post-exposure			After Treatment with PAβN		
Gentamicin *	1.12 (±0.29)	1.29 (±0.26)	+18 (±23)	0.053	1.21 (±0.26)	−6 (±13)	0.346
Chloramphenicol *	6.50 (±1.51)	13.67 (±9.22)	+106 (±135)	0.035	7.83 (±3.35)	−24 (±32)	0.059
Ciprofloxacin *	0.03 (±0.01)	0.11 (±0.09)	+316 (±363)	0.034	0.12 (±0.10)	−1 (±20)	1.0
Trimethoprim/sulphamethoxazole *	0.09 (±0.02)	0.14 (±0.06)	+58 (±55)	0.014	0.12 (±0.05)	−7 (±17)	0.371
Tetracycline *	2.42 (±0.90)	23.29 (±25.98)	+776 (±1027)	0.001	21.92 (±25.02)	−1 (±22)	0.423
Ampicillin **	18.6 (±1.5)	12.0 (±6.4)	+35 (±35)	0.014	11.8 (±6.2)	−1 (±3)	1.0

*MIC (µg/mL); **Diameter of zone inhibition (mm); SD: Standard Deviation.

The changes in MIC or the zone diameter of strains cultured in disinfectant-free media (compared to pre-exposure strains) were −19.9% (SD ± 14.6), −4.2% (SD ± 20.9), −12.8% (SD ± 15.9), +5.5% (SD ± 13.0), +11.8% (SD ± 37.0), and +3.3% (SD ± 5.3) for ciprofloxacin, chloramphenicol, trimethoprim/sulphamethoxazole, gentamicin, tetracycline, and ampicillin, respectively. None of these changes were statistically significant (*p* > 0.05, Wilcoxon tests).

### 2.4. Impact of Efflux Pump Blocking on MIC of Product A and Antimicrobial Resistance

All strains were treated with the generic efflux pump inhibitor phenyl-arginine beta-naphthylamide (PAβN) after the adaptation experiment. This resulted in an overall mean reduction of 18% (SD ± 15) in the MIC for Product A (from 27 μg/mL (SD ± 12)) after exposure to Product A to 21 μg/mL (SD ± 9) after treatment with PAβN) (*p* = 0.04; paired Wilcoxon test) (Table 1). The observed variability in observed MIC changes between experimental replicates was moderate, with a median coefficient of variation of 17.3% (IQR 0–45%).

Treatment with PAβN did not result in significant changes in the susceptibility of antimicrobials with the exception of chloramphenicol (mean reduction of 24% (SD ± 35), *p* = 0.059). A total of four strains (three NTS and one *E. coli*) reverted to being fully susceptible to chloramphenicol after treatment with PAβN (Table 2 and Figure 2). 

### 2.5. Impact of Efflux Pump Blocking on AMR among Representative E. coli Isolates from Animals

After treatment with PAβN, *E. coli* isolates increased their inhibition zone diameters in the disc diffusion test to all investigated antimicrobials (*p* ≤ 0.002, paired Wilcoxon test). The percent of strains that increased their zone diameter by at least 1 mm was greatest for ciprofloxacin (39.3%), followed by chloramphenicol (27.1%), gentamicin (25.2%), ampicillin (16.8%), trimethoprim/sulphamethoxazole (16.8%), and tetracycline (7.7%) (Figure S1).

However, the categorical changes in the observed prevalence of AMR (intermediate and fully resistant taken together) based on CLSI guidelines before and after treatment with PAβN were much more modest. The treatment resulted in reductions in absolute levels of resistance prevalence of 3.3%, 2.6%, 2.6%, and 0.7% for chloramphenicol, ciprofloxacin, ampicillin, and gentamicin, respectively. A total of 7.8% isolates changed their profile from fully resistant to intermediate resistant for ampicillin. No changes in the prevalence of resistance for trimethoprim/sulphamethoxazole and tetracycline were observed (Table 3).

**Table 3:** Prevalence (%) of antimicrobial resistance among 155 representative *E. coli* isolates from animals before and after treatment with efflux pump inhibitor (PAβN).

Antimicrobial	Before Treatment with PAβN				After Treatment with PAβN				Relative Change
	S	I	R	I+R	S	I	R	I+R	
Gentamicin	80.0	5.2	14.8	20.0	80.7	4.5	14.8	19.3	−0.7
Chloramphenicol	43.8	5.2	51.0	56.2	47.1	1.9	51.0	52.9	−3.3
Ciprofloxacin	70.3	12.9	16.8	29.7	72.9	10.3	16.8	27.1	−2.6
Trimethoprim/sulphamethoxazole	40.0	1.3	58.7	60.0	40.0	1.3	58.7	60.0	0
Tetracycline	23.9	0.1	76.0	76.1	23.9	0.1	76.0	76.1	0
Ampicillin	12.9	16.1	71.0	87.1	15.5	21.3	63.2	84.5	−2.6

S: Fully sensitive; I: Intermediate resistant; R: Fully resistant.

## 3. Discussion

This is one of few studies demonstrating an association between increases in MIC for a commercial QAC- and glutaraldehyde-based disinfectant and AMR on field Enterobacteriacea (*E. coli* and NTS) strains. Although the investigated product is widely used on food animal farms in southern Vietnam, the chosen strains originated from healthy human individuals since we expected such isolates have a low chance of previous exposure to disinfectants commonly used on pig and poultry farms.

After a 12-day adaptation period, increases in MIC for a commercial disinfectant product containing a QAC and glutaraldehyde ranged from 0% to +100% (median +31%). Previous adaptation experiments using *E. coli* referent strains gradually exposed to benzalkonium chloride resulted in comparatively greater MIC changes ranging from +500%–600% [10,17]. Another study on a *Salmonella* Virchow isolate resulted in an even greater MIC increase for benzalkonium chloride from 4 μg/mL to 256 μg/mL, *i.e.*, a +6300% increase [18]. Such experiments were carried out using pure benzalkonium chloride; in contrast, in our study we investigated a commercial product that contains a mixture of benzalkonium chloride and glutaraldehyde, and therefore adaptation to either of the two components could theoretically result in an increased MIC for the commercial product. In addition to benzalkonium chloride, the phenomenon of adaptation has also been described with other QACs commonly used in the food industry and animal production such as dimethyl didecyl ammonium chloride (DDAC) and dimethyl dioctyl ammonium chloride (OCDAC) [19].

The observed increases in MIC for the disinfectant product were strongly associated with marked increases in MIC (or decreases in inhibition zone) for all antimicrobials tested. The greatest changes were observed in decreasing order for ampicillin, tetracycline, ciprofloxacin, chloramphenicol, trimethoprim/sulphamethoxazole, and gentamicin. These results are consistent with previous studies investigating cross-resistance between QAC-based disinfectants and a range of antimicrobials [10,17,18,19,20]. Interestingly, our results suggest that the tested product did not contribute to changes in the observed resistance pattern for the aminoglycoside (*i.e.*, gentamicin), which is in agreement with a previous study investigating cross-resistance between QACs and a range of antimicrobials [19]. The fact that an association between a commercial disinfectant and ciprofloxacin was shown is of particular concern since fluoroquinolones are defined as antimicrobials of critical importance for human medicine [21].

Recent studies by our research group on livestock and poultry farms in southern Vietnam showed that ampicillin and tetracycline were the two antimicrobials (of eight tested) against which *E. coli* strains exhibited the highest levels of resistance [14]. Similarly, these two antimicrobials were the first and third (of 10 tested) against which NTS isolates showed the highest resistance levels [15]. Furthermore, *E. coli* from small wild mammals (rats and shrews) trapped on the same farms consistently showed the highest levels of AMR against those two antimicrobials. Since wild mammals are not deliberately treated with antimicrobials, we hypothesize that this may partly be a reflection of co-resistance due to the high volume of disinfectants or antimicrobials that may be discharged into the environment. In contrast to glutaraldehyde, which is readily biodegradable in soil and freshwater [22], QACs are generally considered to have poor biodegradability and are mainly excreted in non-metabolized form [23].

A limitation of our study is the relatively small number of strains investigated. This was dictated by laboratory capacity and the high costs associated with MIC testing for a large number of antimicrobials. In spite of this, the direction of the changes observed (a clear increase in MIC for Product A after the adaptation experiment) and the observed strong association between these changes and the observed increases in MIC for antimicrobials strongly support the phenomenon of cross-resistance. In contrast to small replicate variation, there were considerable differences in MIC results between strains, which could theoretically result in potential biases due the choice of strains.

Treatment of strains with a generic efflux pump inhibitor (PAβN) after the adaptation experiment did not result in significant changes in their MIC for antimicrobials with the exception of chloramphenicol (four strains reverted to full susceptibility). Furthermore, the changes observed after treatment of field strains with PAβN were modest, although in the direction of increased susceptibility. In the case of ampicillin, a significant fraction (7.8%) of field isolates changed their category from fully resistant to intermediate resistant after treatment with PAβN. This is surprising given the low level of reversion of resistance against this antimicrobial observed in Product A-adapted strains after treatment with PAβN. This suggests that the AMR phenotypic expression of induced cross-resistance between the disinfectant product and antimicrobials may be due to mechanisms other than generic efflux pump expression such as gene mutations conferring inactivation of porins or overexpression of glycolitic enzymes [10,20]. Alternatively, given the potential huge diversity of substrate binding sites present in efflux pumps [24] and the lack of information about the specific efflux pumps in our strains, the use of a single universal inhibitor such as PAβN may not have been sufficient to completely block the efflux pump activity in the bacterial cell [25].

In the Mekong Delta of Vietnam over 80% of poultry farms routinely use QAC-based disinfectants, and 74% of farms surveyed use the commercial product tested here, most of them on occupied buildings (Table S1). This is a concern, since in the presence of animal waste disinfectants may be diluted, causing bacterial populations to be exposed to sub-optimal doses for long periods of time, therefore promoting the rapid development of resistance [26].

## 4. Material and Methods

### 4.1. Study Design

We investigated pan-susceptible Enterobacteriaceae strains of human origin that were gradually exposed to increasing concentrations of a benzalkonium chloride- and glutaraldehyde-containing commercial Product A over a 12-day period. Strains were investigated for their antimicrobial susceptibilities against gentamicin, chloramphenicol, ciprofloxacin, trimethoprim/sulphamethoxazole, tetracycline, and ampicillin before and after the adaptation period. The potential induction of efflux pumps in disinfectant/antimicrobial cross-resistance mechanisms was investigated by incubating bacterial strains after the adaptation experiments with PAβN, a generic efflux pump inhibitor and subsequent AMR testing. We also treated with PAβN a sample of representative *E. coli* isolates of farm animal origin to investigate the overall potential contribution of efflux pumps on observed levels of AMR.

### 4.2. Selection of Strains and AMR Testing

Six *E. coli* and six NTS strains were used. Of those, 10 isolates (five *E. coli* and five NTS strains) were obtained from healthy human subjects as part of a larger study investigating AMR in humans and animals in Tien Giang Province (Vietnam). In addition, two fully susceptible laboratory strains, *Salmonella* Typhimurium 14028 and *E. coli* 25922, were included for quality control of susceptibility tests*. E. coli* isolates were confirmed using standard biochemical tests (motility, indole, lactose/glucose fermentation, methyl red, citrate, urease, hydrogen sulfide, and gas production). NTS were isolated using selective media (Rambach and Xylose Lysine Deoxycholate agar) and confirmed by serological method (PSO and PSH polyvalent). All selected strains were sensitive against the six antimicrobials investigated using the Bauer-Kirby disc diffusion method according to Clinical and Laboratory Standard Institute (CLSI) procedures [27]: gentamicin (CN, 10 μg), chloramphenicol (C, 30 μg), ciprofloxacin (CIP, 5 μg), trimethoprim/sulphamethoxazole (SXT, 1.25/23.75 μg), tetracycline (TE, 30 μg), ampicillin (AMP, 10 μg) (Oxoid, Hampshire, UK). Etest strips (BioMerieux, Marcy I’Etoile, France) were used to determine the minimum inhibitory concentration (MIC) for gentamicin (GM256), chloramphenicol (CL256), ciprofloxacin (CI32), trimethoprim/sulphamethoxazole (TS32), and tetracycline (TC256). Since the Etest of ampicillin was not available in our laboratory, AMR for ampicillin was tested using disc diffusion method (AMP, 10 μg).

### 4.3. Adaptation Experiment to Product A

Product A, a commercial disinfectant containing benzalkonium chloride (150 mg/mL) and glutaraldehyde (150 mg/mL) as main active ingredients, was used in the adaptation experiment.

The baseline MIC for Product A corresponded to the lowest concentration of the product that prevents bacterial growth after incubation for 24 h at 37 °C. The obtained MIC represents the concentration of active components (both benzalkonium and glutaraldehyde). The MIC was first determined for each strain using a serial dilution method: from stock solution of Product A, serial dilutions ranging from 60 to 300 μg/mL in distilled water were prepared. 200 μL of each dilution was added to 1.8 mL of MH broth (Oxoid, Hampshire, UK) containing a bacterial suspension with 10^5^ CFU/mL and incubated for 24 h at 37 °C. Bacterial growth was assessed by plating into MH agar (Oxoid, Hampshire, UK), followed by incubation for 24 h.

The adaptation experiment was performed according to a previously described procedure [19]. Briefly, colonies were suspended in MH broth and incubated for 24 h at 37 °C. An aliquot of 50 µL of a calibrated suspension containing 10^8^ CFU/mL of each strain was initially mixed with an initial concentration of 0.5 MIC of Product A in a total volume of 5 mL of MH broth, and incubated for 24 h at 37 °C. After incubating, cultures were calibrated to concentration of 10^8^ CFU/mL. Then 50 μL of suspension was transferred to 5 mL of MH broth containing higher concentration of disinfectant. The cultures were also plated onto MH agar for growth confirmation and storage. The procedure was repeated daily over 12 days or stopped earlier if no growth was observed. The highest concentration of Product A in which bacteria could grow was used. The observed change in MIC before and after the adaptation experiment was recorded in absolute terms (µg/mL) for each experimental replicate. The reported MIC to Product A before and after adaptation was calculated as the average of the three experimental replicates. In order to exclude the potential development of MIC changes due to spontaneous mutation as a result of exposure to disinfectant-free nutrient broth, all 12 strains were tested for their MIC of Product A after a 12-day period in MH broth at 37 °C. All strains were investigated in triplicate.

### 4.4. Investigation of the Changes in AMR before and after Exposure to Product A

For all 12 *E. coli* and NTS strains the MIC changes (gentamicin, chloramphenicol, trimethoprim/ sulphamethoxazole, ciprofloxacin, tetracycline) as well as zone diameter change (ampicillin) were investigated before and after the adaptation experiment. In addition, potential changes in the AMR profile (MIC and zone diameter) due to exposure to disinfectant-free nutrient broth was investigated among all studied strains after a 12-day period in MH broth at 37 °C.

### 4.5. Investigation of the Role of Efflux Pumps on Antimicrobial Resistance

The potential contribution of efflux pumps on the observed pattern of resistance was investigated by treating all 12 studied strains with 50 μg/mL of the generic efflux pump inhibitor phenyl-arginine beta-naphthylamide (PAβN) (Sigma, Munich, Germany) [25] as described elsewhere [28]. The observed changes in MIC before and after treatment with PAβN for each experimental replicate were recorded in absolute terms (µg/mL). The reported MIC to Product A before and after adaptation was calculated as the average of the three experimental replicates. In addition, a selection of 155 representative *E. coli* isolates from chicken, duck, pig farms and small wild mammals trapped on farms and rice field/forest sites from the Mekong Delta region of Vietnam [14] were tested for AMR (disc diffusion method) before and after treatment with 50 μg/mL of PAβN.

### 4.6. Statistical Analyses

Changes in the MIC for Product A before and after the adaptation experiment and after treatment with PAβN were compared using the non-parametric paired Wilcoxon signed-rank test. The potential statistical association between observed changes in MIC for Product A and AMR as a result of the adaptation experiments was investigated using Pearson’s correlation test. Kruskal-Wallis paired tests were used to compare MIC for Product A between *E. coli* and NTS before and after the adaptation experiment. Analyses were carried out using the “epicalc” package in R software (http://www.r-project.org/).

## 5. Conclusions

We conclusively demonstrated *in vitro* the phenomena of cross-resistance between benzalkonium chloride- and glutaraldehyde-based disinfectant and antimicrobials. The data suggests that only a small fraction of the observed AMR may be due to generic efflux pumps and more research is required to determine the molecular mechanisms responsible for these changes. Given the widespread and often inadequate use of disinfectants on farms in Vietnam, we suggest that this could potentially be a factor contributing to the observed high levels of AMR among Enterobacteriaceae. We recommend performing well-planned longitudinal studies to investigate molecular changes in organisms before and after exposure to such disinfectants. We also strongly recommend better training on the correct use of disinfectants on farms, since preserving the efficacy of disinfectants is crucial to maintaining hygiene levels on farms and reducing the need for using antimicrobials.

## Supplementary Files

Supplementary File 1
