# Supplementary material for: Induction of Antimicrobial Resistance in Escherichia coli and Non-Typhoidal Salmonella Strains after Adaptation to Disinfectant Commonly Used on Farms in Vietnam
Source: Antibiotics (Basel). 2015 Oct 30;4(4):480–94. doi: 10.3390/antibiotics4040480 (PMC4790309; doi:10.3390/antibiotics4040480)
Supplement: Supplementary File 1 [file antibiotics-04-00480-s001.pdf]

Supplementary Material

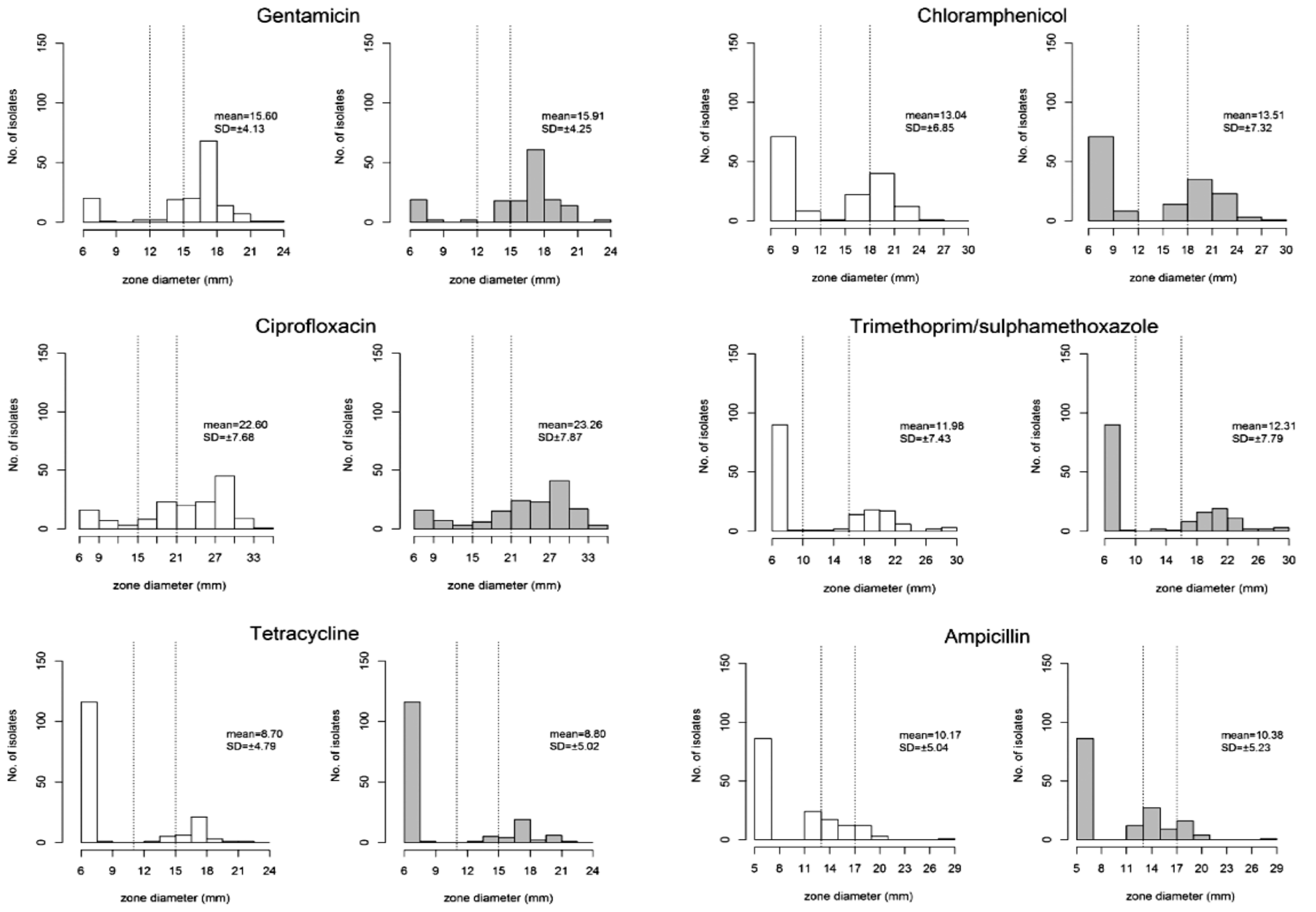

Figure S1. Distribution of inhibition zone diameter (mm) among 155 representative *E. coli* strains for 6 antimicrobials investigated before and after treatment with PAβN. White bars: before treatment with PAβN. Grey bars: after treatment with PAβN. Dotted lines: breakpoints for susceptible, intermediate and resistant classes based on CLSI guidelines.

Table S1. Most common disinfectant products used for disinfection in 208 chicken farms in Tien Giang province, Mekong Delta, Vietnam (2012–2013).

Product	Main Components	Proportion of Farms Using over 12 months (%)
A	Benzalkonium chloride<br>Glutaraldehyde	74.0
B	DDAC, DODAC, ODDAC, ADAC *<br>Glutaraldehyde	28.4
C	PVP Iodine	8.1
D	Potassium monopersulphate	5.8
E	PVP Iodine<br>Glycerin	4.8
Others		21.6

* DDAC: Didecyl dimethyl ammonium chloride; DODAC: Dioctyl dimethyl ammonium chloride; ODDAC: Octyl decyldimethyl ammonium chloride; ADAC: Alkyl dimethylbezyl ammonium chloride.

© 2015 by the authors; licensee MDPI, Basel, Switzerland. This article is an open access article distributed under the terms and conditions of the Creative Commons Attribution license (http://creativecommons.org/licenses/by/4.0/).
